# *Kokumi* Substances, Enhancers of Basic Tastes, Induce Responses in Calcium-Sensing Receptor Expressing Taste Cells

**DOI:** 10.1371/journal.pone.0034489

**Published:** 2012-04-12

**Authors:** Yutaka Maruyama, Reiko Yasuda, Motonaka Kuroda, Yuzuru Eto

**Affiliations:** Institute for Innovation, Ajinomoto Co., Inc., Kawasaki, Japan; German Institute for Human Nutrition, Germany

## Abstract

Recently, we reported that calcium-sensing receptor (CaSR) is a receptor for *kokumi* substances, which enhance the intensities of salty, sweet and umami tastes. Furthermore, we found that several γ-glutamyl peptides, which are CaSR agonists, are *kokumi* substances. In this study, we elucidated the receptor cells for *kokumi* substances, and their physiological properties. For this purpose, we used Calcium Green-1 loaded mouse taste cells in lingual tissue slices and confocal microscopy. *Kokumi* substances, applied focally around taste pores, induced an increase in the intracellular Ca^2+^ concentration ([Ca^2+^]_i_) in a subset of taste cells. These responses were inhibited by pretreatment with the CaSR inhibitor, NPS2143. However, the *kokumi* substance-induced responses did not require extracellular Ca^2+^. CaSR-expressing taste cells are a different subset of cells from the T1R3-expressing umami or sweet taste receptor cells. These observations indicate that CaSR-expressing taste cells are the primary detectors of *kokumi* substances, and that they are an independent population from the influenced basic taste receptor cells, at least in the case of sweet and umami.

## Introduction

The extracellular calcium-sensing receptor, CaSR, is a classic seven-transmembrane-spanning, G protein-coupled receptor (GPCR) belonging to Family C of the superfamily of GPCRs [1]. CaSR has been identified in several cells and tissues, including the parathyroid gland and kidney. It plays a central role in extracellular calcium homeostasis in mammals [2]. An increase in the blood calcium level is sensed by CaSR, which in turn suppresses parathyroid hormone secretion, stimulates calcitonin secretion, and induces urinary calcium excretion to reduce blood calcium to normal levels. It has become apparent that CaSR is expressed not only in the parathyroid glands and kidney, but also in many other tissues such as liver, heart, lung, gastrointestinal tract, pancreas and the central nervous system, suggesting that it is involved in a range of biological functions [3]. It has been reported that CaSR is activated by several types of substances including cations such as Ca^2+^, Mg^2+^ and Gd^3+^, basic peptides such as protamine and polylysine, and polyamines such as spermine [3].

CaSR is expressed in a subpopulation of taste cells in mice and rats [4], [5], suggesting potential roles for this receptor in taste cellular biology. Ninomiya and colleagues reported that mice have a group of gustatory afferent nerve fibers that respond to calcium and magnesium [6]. Tordoff and co-workers described the taste perception of calcium and the physiological mechanisms underlying calcium intake, appetite and homeostasis, and indicated that calcium deprivation increases the palatability of calcium [7]. These findings indicate the existence of a calcium transduction mechanism in taste cells. However, except for calcium, the physiological role of these CaSR agonists is not clear. Recently, Bystrova *et al*. reported that CaSR is expressed in a subset of taste cells, and that the agonists, NPS R-568, neomycin and several L-amino acids, induced a response in isolated taste cells [4].

Recently, we reported that various CaSR agonists including γ-glutamyl-cysteinyl-glycine (reduced form of glutathione, GSH) and other γ-glutamyl peptides have enhancing activities on umami, sweet and salty tastes, and that there is a high correlation between CaSR agonist activity and taste intensity [8]. Ueda *et al*. reported that water extracts from garlic, which contain GSH, enhance umami taste intensity, and they propounded the taste enhancing character as the “*kokumi* flavor” [9]–[12]. Furthermore, we identified several γ-glutamyl peptides, which are CaSR agonists that have a *kokumi* flavor activity, and found that γ-glutamyl-valinyl-glycine (γEVG) is the most potent *kokumi* substance [8]. These results suggest that CaSR-expressing taste cells in lingual epithelium respond to *kokumi* substances.

In the present study, we employed a semi-intact lingual slice preparation in which it is possible to focally apply *kokumi* stimuli onto the apical tips of the taste buds and measure individual cellular responses with enough time and spatial resolution for Ca^2+^ imaging. We show that *kokumi* substances induce a [Ca^2+^]_i_ response in taste cells in the posterior tongue. The results indicate that *kokumi* substances are detected by CaSR-expressing taste cells.

## Results

### *CaSR* is expressed in the taste buds in lingual epithelia

**Figure 1:**
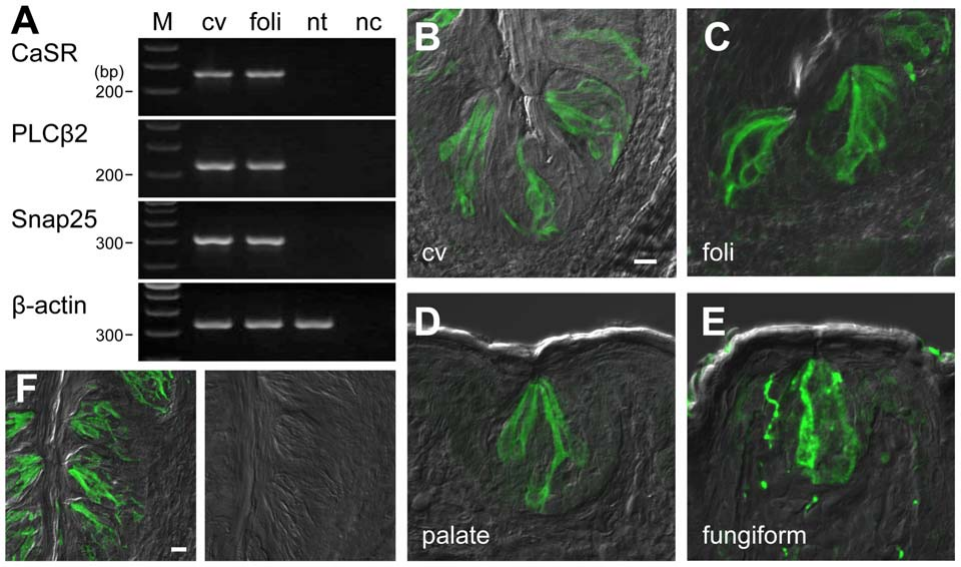
Taste cells express CaSR. (A) RT-PCR for *CaSR* expression in taste bud-enriched circumvallate (cv), foliate (foli) and non-taste bud (nt) lingual epithelium. nc - negative control (lacking template); M - molecular standard. (B–E) Immunostaining for CaSR in taste buds. CaSR immunofluorescence is seen in most circumvallate (B), foliate (C), palate (D) and fungiform (E) taste buds. Immunofluorescent images (green) were superimposed on DIC images. (F) Validating the anti-CaSR antibody. The CaSR antiserum was preabsorbed with an excess of antigen peptides. The circumvallate sections reacted with preabsorbed and non-absorbed antibodies and were processed simultaneously. Images were taken under the same illumination conditions and detector settings. Scale bars 20 µm.

**Figure 2:**
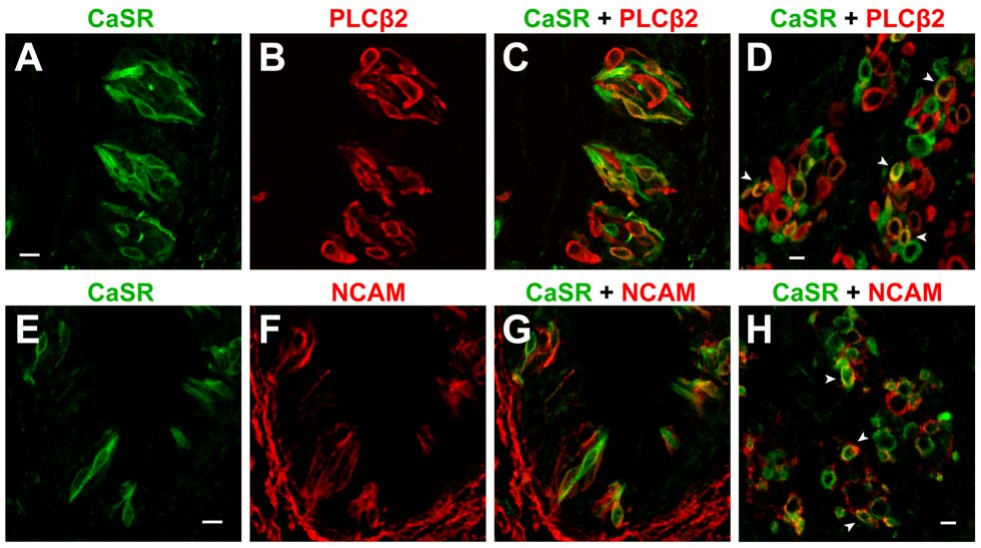
Confocal images showing colocalization of CaSR and the taste cell markers in taste cells from mouse circumvallate papillae. (A–C) A longitudinal section of a circumvallate taste bud immunostained with antibodies against CaSR (A) and PLCβ2 (B). (C) Overlay of A and B. (D) A transverse section of a circumvallate taste bud immunostained with antibodies against CaSR (green) and PLCβ2 (red). (E–G) A longitudinal section of a circumvallate taste bud immunostained with antibodies against CaSR (E) and NCAM (F). (G) Overlay of E and F. (H) A transverse section of a circumvallate taste bud immunostained with antibodies against CaSR (green) and NCAM (red). Scale bars 20 µm. Arrowheads indicate double-labeled cells.

We tested the expression of *CaSR* mRNA in taste buds and in non-taste lingual epithelium from a C57BL/6 mouse by RT-PCR. We confirmed that *CaSR* mRNA was expressed in circumvallate and foliate, but not in non-taste epithelium (Fig. 1A). To determine the presence of CaSR in taste cells, we employed immunohistochemistry on mice lingual tissues. CaSR immunoreactivity was observed in a subset of spindle-shaped taste cells in circumvallate, foliate, fungiform and palate papillae (Fig. 1B–E). In the transverse section of circumvallate taste buds, 8–10 CaSR-immunoreactive taste cells were present in a taste bud (Fig. 2D, H). The specificity of the antibody was confirmed by antigen preabsorption, which resulted in little or no immunoreaction in taste cells (Fig. 1F).

### *CaSR* is expressed in a subset of type II (receptor) and type III (presynaptic) cells

Mammalian taste buds contain three distinct classes of cells [13]–[15]. A heterogeneous population of mammalian taste cells includes morphologically and functionally different taste cells classified into three subtypes, type I (glial-like cells), type II (receptor cells) and type III (presynaptic) taste cells [16]. These classes express different complements of genes related to their functions: receptor (Type II) cells express G-protein coupled taste receptors and transduction machinery. In contrast, presynaptic (Type III) cells express neuronal proteins, including those associated with synapses, and also respond to sour stimuli [17]–[21].

To characterize the CaSR-immunoreactive taste cells, we investigated the coexpression of CaSR and taste cell markers. Using immunofluorescence, previous reports have shown in circumvallate taste buds the expression of PLCβ2 in type II taste cells (receptor cells) [17], and of neural cell adhesion molecule (NCAM) in type III taste cells (presynaptic cells) [22]. Consequently, we used double immunofluorescence microscopy to evaluate whether these CaSR-expressing taste cells co-expressed taste cell markers. Using immunohistochemistry, we observed that the CaSR-expressing taste cells also expressed either PLCβ2 or NCAM (Fig. 2). Out of 728 CaSR-positive cells, 314 cells expressed PLCβ2 (43.1%; Fig. 2D), while the other CaSR-positive cells expressed NCAM (669 cells out of 1033 cells, 64.7%; Fig. 2H). Conversely, out of 823 PLCβ2-positive cells, 314 cells expressed CaSR (38.2%). These populations were in agreement with previous findings regarding the cell-types of CaSR-expressing taste cells [5].

### Taste cells respond to focally applied *kokumi* substances

**Figure 3:**
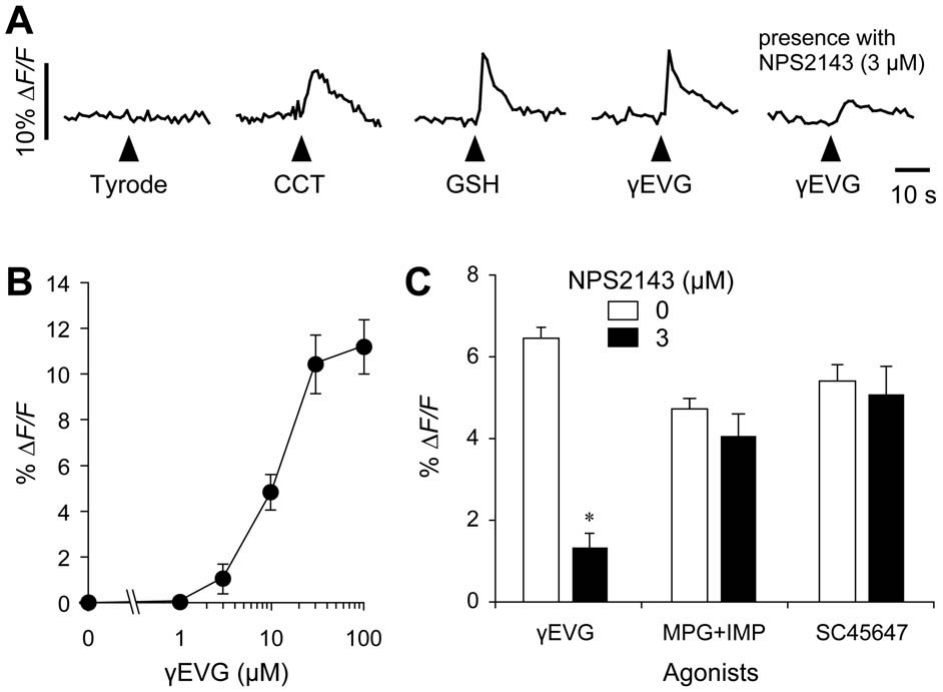
Taste cell responses (ΔCa^2+^) evoked by *kokumi* stimuli, recorded in a slice preparation of the mouse circumvallate papilla. (A) Taste cells were stimulated sequentially with three *kokumi* substances, cinacalcet (CCT, 10 µM), glutathione (GSH, 100 µM) and γ-glutamyl-valinyl-glycine (γEVG, 100 µM), as well as γEVG + NPS2143 (3 µM), a CaSR antagonist. Arrowheads below traces indicate the stimulation. (B) Concentration-response relationship for γEVG (mean ± SE; n = 4 cells). (C) Taste responses elicited by γEVG (100 µM) were inhibited by the CaSR antagonist, NPS2143 (3 µM), but the umami (MPG 100 mM + IMP 1 mM) and the sweet (SC45647, 10 µM) responses were unaffected. Mean amplitudes of γEVG-, MPG + IMP-, and SC45647-induced responses in the presence or absence of 3 µM NPS2143 are shown (mean ± SE; *p≤0.05, n = 4 cells). Raw traces are shown in A.

To test whether *kokumi* substances induce an intracellular Ca^2+^ response in taste cells and if so, to identify which taste cells are responsible, we employed semi-intact lingual slice preparations, focally applied *kokumi* substances to the apical chemosensory tips of the taste cells, and imaged the intracellular [Ca^2+^] changes in the taste cells with confocal scanning microscopy [23], [24]. Focal application of the *kokumi* substances, cinacalcet (a classic CaSR agonist; 10 µM), glutathione (GSH; 100 µM), or γ-glutamyl-valinyl-glycine (γEVG; 100 µM), induced Ca^2+^ responses (Δ[Ca^2+^]_i_) in a small fraction of the taste cells (Fig. 3A) [8]. These responses were not induced by solution puffing, because no response was observed after ejection of Tyrode's solution (Fig 3A). Some, but not all, *kokumi* substance-responsive cells also responded to bath applied KCl (50 mM), which induces a Ca^2+^ response in the presynaptic (Type III) taste cells via depolarization of the plasma membrane. γEVG evoked a transient [Ca^2+^]_i_ increase in 6.5% of the taste cells in the circumvallate papilla (34 of 524 cells obtained from 26 mice). The mean amplitude of Ca^2+^ responses (Δ*F/F*) evoked by 100 µM γEVG was 11.6±1.7% (mean ± SE; n = 21 cells; Fig. 3B). The EC_50_ value of γEVG was estimated at approximately 13 µM (Fig. 3B). Importantly, applying higher concentrations of γEVG (>30 µM) did not induce Ca^2+^ responses in additional taste cells. Furthermore, *kokumi* substance-induced responses were selectively blocked by 3 µM NPS2143, a CaSR inhibitor, which barely affected the umami (MPG 100 mM + IMP 1 mM) and the sweet (SC45647, 100 µM) responses (Fig. 3C) [8], [25]. This suggests that the Ca^2+^ responses we recorded reflect selective stimulation of a specific subpopulation of *kokumi* substance-responsive taste cells.

### Responses to *kokumi* substance stimulus involve Ca^2+^ release

**Figure 4:**
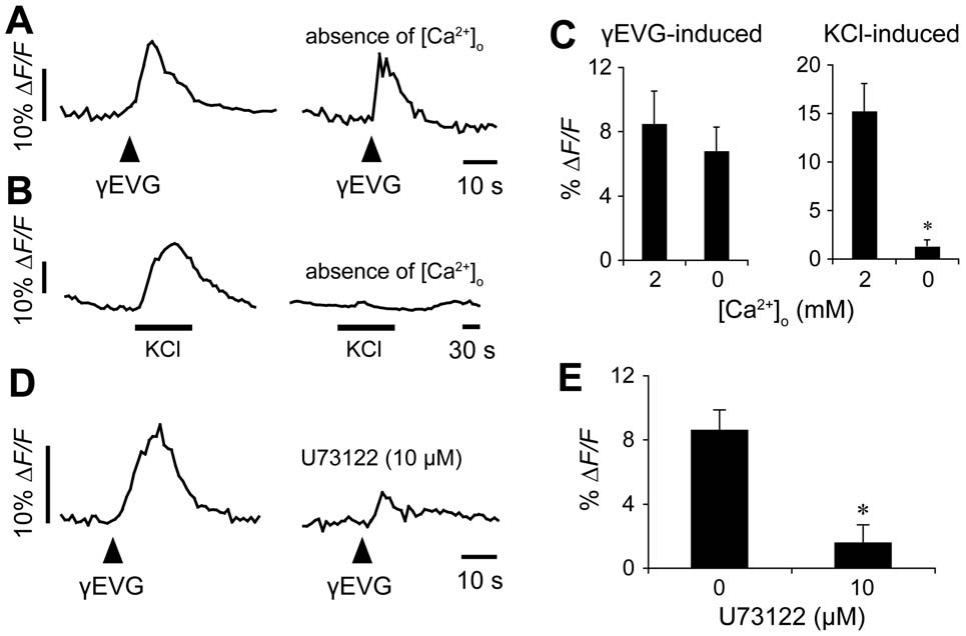
Ca^2+^ response elicited by γEVG involves intracellular Ca^2+^ stores and phospholipase C. (A) γEVG (100 µM) was focally applied in medium containing Ca^2+^ (left trace) or in the absence of Ca^2+^ (Ca-free medium with 0.2 mM EGTA; right trace). (B) Responses elicited by depolarization (bath-applied KCl, 50 mM) and influx of Ca^2+^ through voltage-dependent Ca^2+^ channels were abolished in the absence of extracellular Ca^2+^. (C) Mean amplitudes of responses in the presence or absence of Ca^2+^ in the medium (mean ± SE; *p≤0.05, n = 4 cells). (D) Responses to γEVG inhibited by U73122 (10 µM). (E) Mean amplitudes of the responses in the presence or absence of U73122 (mean ± SE; *p≤0.05, n = 4 cells).

Next, we investigated the Ca^2+^-mobilizing pathway that is activated by the *kokumi* substance stimulus in mouse circumvallate taste cells. We examined responses in acute absence of extracellular Ca^2+^ by bathing slices in a Ca^2+^-free Tyrode's solution (containing 0.2 mM EGTA) 2 min before the focal *kokumi* substance stimulation. As shown in Fig. 4A, responses evoked by γEVG did not significantly change compared with the presence of extracellular Ca^2+^ (control, Δ*F/F* = 7.1±1.8%; Ca^2+^-free, Δ*F/F* = 6.7±2.0%; n = 5). In contrast, the depolarization-evoked Ca^2+^ response, elicited by perfusing the slice with 50 mM KCl that allows Ca^2+^ influx through voltage-gated Ca^2+^ channels in Type III presynaptic taste cells [26], was nearly completely abolished in the absence of extracellular Ca^2+^ under parallel treatments (control, Δ*F/F* = 16.1±3.6%; Ca^2+^-free, Δ*F/F* = 2.8±0.2%; *n* = 4; Fig. 4B). Part of the γEVG-responsive taste cells showed a Ca^2+^ response to KCl stimulation; however, the γEVG-induced response in these cells was not affected by Ca^2+^-free conditions (Fig. 4A and B). These results are consistent with *kokumi* transduction involving release of stored Ca^2+^, not Ca^2+^ influx (Fig. 4C).

Umami, sweet and bitter stimuli trigger the release of stored Ca^2+^ by activating phospholipase C (PLC) [24], [27]–[30]. To directly test whether *kokumi* substance-elicited Ca^2+^ responses arise from PLC activation, we used a non-selective PLC inhibitor, U73122 [31]–[33]. After incubation with 10 µM U73122 for 10 min, responses elicited by γEVG were almost abolished (control, Δ*F/F* = 8.3±1.6%; U73122, Δ*F/F* = 1.7±0.8%; *n* = 4; Fig. 4D and E). In contrast, depolarization (KCl)-induced responses were not significantly altered by treatment with U73122 (data not shown). These data strongly support the notion that the *kokumi* mechanisms involve intracellular Ca^2+^ release.

### *CaSR* ligand-responsive cells do not respond to L-glutamate stimuli

**Figure 5:**
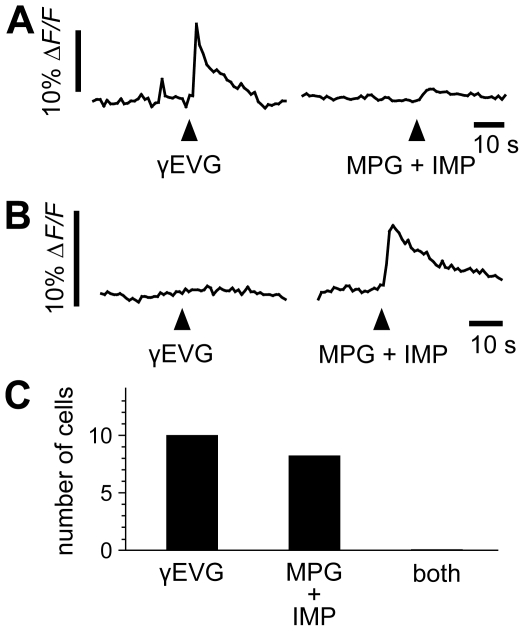
γEVG-responding taste cells are different from MPG + IMP-responding cells. (A) Taste cell responses were recorded in lingual slice preparations of mouse circumvallate papilla. Preparations were sequentially stimulated with γEVG (100 µM) and MPG (100 mM) + IMP (1 mM). The traces show superimposed responses from γEVG-responding cells (A) and MPG + IMP-responding cells (B). Responses to γEVG were only observed in cells lacking response to MPG + IMP. (C) We recorded from 132 Calcium Green-loaded taste cells in 16 lingual slices. Ten dye-loaded cells were γEVG-responding cells, while MPG + IMP stimulation evoked responses in 8 out of 132 cells. We did not identify cells that responded to both substances.

It has been reported that CaSR is activated by various γ-glutamyl peptides, including glutathione and γEVG [8], [34], [35]. When transiently expressed in HEK293 cells, CaSR also induces Ca^2+^ changes in response to L-glutamate monomer, which is associated with the umami taste [4]. In contrast, receptor (Type II) cells respond to sweet, bitter and umami taste stimuli by elevating cytoplasmic Ca^2+^ [17], [21]. We asked whether the CaSR ligand, γEVG, and glutamate produce responses in the same mouse type II taste cells. These studies have suggested that γEVG mimics the taste of L-glutamate, at least in part, by activating the same taste receptors as umami compounds. To test this interpretation directly, we focally applied γEVG and monopotassium L-glutamate (MPG) + inosine monophosphate (IMP) sequentially on circumvallate taste buds. γEVG (100 µM) evoked transient Ca^2+^ responses in some taste cells (10 responding cells out of 132 recorded cells; Δ*F/F* = 7.4±1.3%), but not in those that responded to MPG (100 mM) + IMP (1 mM) (Fig. 5A, C). Conversely, MPG + IMP-responding cells (8 cells out of 132 recorded cells; Δ*F/F* = 6.7±1.5%) did not respond to γEVG (Fig. 5B, C). These data suggest that separate receptors, found on separate cells, generate Ca^2+^ responses to γEVG and MPG + IMP (Fig. 5C). In cells that respond to each agonist, we cannot rule out the possibility of subthreshold responses to the other agonist. Nevertheless, these results emphasize that responses to CaSR ligand in native taste tissues are highly heterogeneous and vary markedly from those described for the proposed umami taste receptors.

### Physiological responses correlate with molecular expression

**Figure 6:**
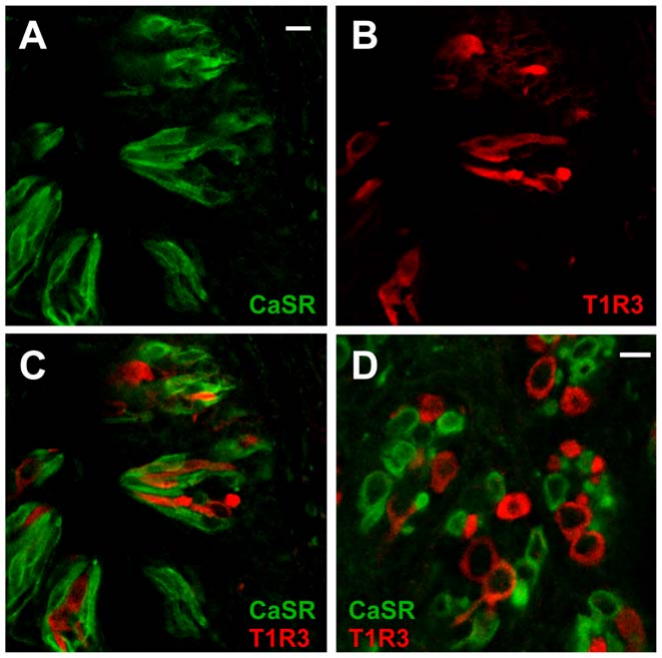
CaSR is found in distinct cells that do not express an umami/sweet receptor subunit. (A–C) A longitudinal section of a circumvallate taste bud immunostained with antibodies against CaSR (A) and T1R3 (B). (C) Overlay of A and B. (D) A transverse section of a circumvallate taste bud immunostained with antibodies against CaSR (green) and T1R3 (red). Scale bars 20 µm.

We recorded Ca^2+^ responses to 100 µM γEVG and 100 mM MPG in lingual slice preparations as described above. The functional responses of taste cells in lingual slices fell into two distinct classes. Our next step was to test whether the two kinds of responding taste cells, determined by functional imaging, mapped onto the two categories determined by expression of CaSR and an umami receptor subunit, T1R3 [36]. We designed additional methods, which are independent from the methods described above, to distinguish between CaSR-expressing cells and T1R3 cells. To identify these receptors, we used dual immunohistochemistry for CaSR and T1R3 in mouse circumvallate papillae. Examples of immunostained circumvallate papilla are shown in Fig. 6. The presence of CaSR immunofluorescent signals was observed in a subset of taste cells. In 63 circumvallate taste buds, 502 taste cells expressed CaSR, whereas 347 taste cells expressed T1R3. Only three cells (0.6% of CaSR-positive taste cells) expressed both CaSR and T1R3 (Fig. 6). These data demonstrate that most taste cells express CaSR, T1R3, or neither.

## Discussion

In this study, we used a preparation of lingual slices from circumvallate papilla that allows one to apply tastants selectively to the apical chemosensory tips of taste cells, while avoiding stimulating non-taste cells and basolateral regions of taste buds [24]. This report presents data that indicate that CaSR ligands induce a cellular response in mouse circumvallate taste cells. We identified a population (6.5%) of taste cells in the circumvallate papillae that responds to *kokumi* substances. This value is comparable with the immunohistochemistry results (6.8% CaSR-positive cells/taste bud). Furthermore, this compares with previous estimates, in the same preparation, of 28% cells that respond to bitter [37] and 5% to umami (monopotassium L-glutamate) stimuli [24]. The threshold concentration of γEVG needed to activate a Ca^2+^ response in taste cells was 3 µM, which is consistent with reports on human sensory evaluation analysis [8]. We also observed that both species respond to CaSR agonists with very similar characteristics (Figure S1, Method S1). Hence, the results from mouse experiments can be extrapolated to results from human CaSR experiments. Additionally, NPS2143, a CaSR inhibitor, impaired the CaSR agonist-induced taste cell response in lingual slice preparations, as well as in heterologous experiments. Moreover, taste cells responded to multiple CaSR agonists, including hydrophobic (cinacalcet) and hydrophilic (GSH and γEVG) *kokumi* substances. Taste buds contain tight junctions around the taste pore (e.g., claudin 4 and 8), and maintain a highly specific permeability barrier for paracellular diffusion [38]. It is expected that CaSR agonists (*kokumi* substances), especially the hydrophilic GSH and γEVG, are accessible only to microvilli in the apical tip of the taste cells, and activate CaSR. Taken together, the data from the present study suggest that CaSR is involved in taste transduction in mice.

Human sensory analysis demonstrated that CaSR agonists enhance the intensity of umami and sweet tastes, and are called *kokumi* flavor [8]. We expected that CaSR agonists would induce a response in the umami or sweet taste cells. Tordoff and colleagues reported that calcium elicits appetitive behavior in mice [7]. Their recent study suggested that CaSR dimerizes with the sweet and umami receptor subunit T1R3 [39]. However, surprisingly, CaSR agonists induced a response in a different group of taste cells from the umami or sweet taste cells. Results from double immunohistochemistry of CaSR and T1R3 also support this observation. Signaling in taste cells, especially for umami, sweet and bitter, are well understood. Taste receptors for umami (T1R1 + T1R3) and sweet (T1R2 + T1R3) agonists have been identified [36], [40]–[42], and activation of these receptors elicits cellular responses (e.g. transient Ca^2+^ changes and ATP secretion) in receptor-expressing taste cells. These findings demonstrate the involvement of CaSR in *kokumi* signaling, and suggest that CaSR is not directly involved in umami or sweet taste signaling. In cells that respond to each agonist, we cannot rule out the possibility of cell-to-cell signaling within a taste bud.

Our results indicate that *kokumi* substance-responsive taste cells are both presynaptic (Type III) and non-presynaptic taste cells. Furthermore, the results suggest that certain stimuli evoke the Ca^2+^ response via release from intracellular stores in both presynaptic and non-presynaptic taste cells. The exact classification of *kokumi* substance-responsive taste cells remains to be determined. San Gabriel *et al*. reported that CaSR-positive taste cells coexpress PLCβ2, a receptor (Type II) cell marker, or NCAM, a presynaptic cell marker in rat vallate taste buds [5]. Taken together with these observations, CaSR is expressed at least in Type II and Type III taste cells. Activation of *kokumi* substance-responsive cells may modulate the activity of sensory afferent fibers, and/or neighboring taste cells in the same taste bud [24], [43], [44]. ATP released from receptor cells may excite primary sensory afferent fibers [45]. ATP may also function as a paracrine transmitter and act on cells within the taste bud [43], [46], [47]. Our experiments were not designed to distinguish between these possibilities, both of which remain open questions.

Recently, Bystrova *et al.* reported that only type III taste cells responded to agonists of CaSR. However, in our study, all CaSR agonists tested induced responses in both type II and type III taste cells. We do not know the reason for this discrepancy; enzyme treatment during taste cell isolation might affect cellular responses. In PLCβ2-positive type II taste cells, CaSR is expressed in T1R3-negative taste cells. Whether CaSR is expressed in bitter receptor T2R-positive cells remains unresolved.

Our results suggest that *kokumi* substance-induced increases in [Ca^2+^]_i_ arise principally via mobilization from intracellular stores, because responses were essentially unaffected by depletion of extracellular Ca^2+^. Conversely, responses were strongly abolished by pre-treatment with U73122, demonstrating the involvement of phospholipase C in *kokumi* transduction. These results provide direct evidence of the existence of a functional *kokumi* receptor coupled to Ca^2+^ mobilization in taste cells. Generally, [Ca^2+^]_i_ response in taste cells seems to depend on two sources of Ca^2+^. For depolarizing stimuli (KCl), Ca^2+^ influx is induced via activation of voltage-gated Ca^2+^ channels in presynaptic taste cells [17], [44], [48]. Interestingly, neither thapsigargin nor U73122 totally eliminated the Ca^2+^ responses evoked by *kokumi* stimuli. Whether this represents minor additional pathways remains unresolved.

In summary, our results demonstrate that *kokumi* substance-responsive cells are presynaptic and non-presynaptic taste cells. We observed that part of the *kokumi* substance-responsive cells did not respond to umami (MPG) or sweet (SC45647) stimuli. The *kokumi* flavor is defined as an enhancer of umami and sweet tastes. *Kokumi* substance-responding taste cells might involve enhancement of these basic tastes.

## Materials and Methods

### Tissue preparation and functional imaging

All experimental procedures were approved by the Animal Experiment Institution Review Board of Ajinomoto Co., Inc., Institute for Innovation (2008220, 2009085, 2010013 and 2011239), and conformed with the standards for the use of laboratory animals published by the Institute of Laboratory Animal Resources, U.S. National Academy of Sciences. C57BL/6 adult mice (≥7 weeks old, male) were sacrificed by exposure to diethyl ether, followed by cervical dislocation. Tongues were removed and immersed in cold Tyrode's solution. Lingual slices containing the vallate papilla were obtained, and a Ca^2+^ indicator dye was injected into taste cells following similar procedures previously described by Maruyama *et al*. [24]. Briefly, the fluorescent Ca^2+^ indicator dye, Calcium Green-1 dextran (CGD; MW 3,000; 0.25 mM in H_2_O; Invitrogen, Carlsbad, CA, USA), was injected iontophoretically through a large-diameter-tip glass micropipette (40 µm) into the crypt surrounding the vallate papilla (−3.5 µA square pulses, 10 min). The CGD-loaded tissue was sliced at 100 µm with a vibratome (Leica VT1000S, Nussloch, Germany). Slices containing vallate taste buds were mounted on a glass coverslip coated with Cell-Tak (Becton Dickinson, Franklin Lakes, NJ, USA), put in a recording chamber, and superfused with Tyrode's solution (30°C) at a rate of 1.5 ml/min. Single glass micropipettes (2 µm tip diameter) were used to directly deliver taste stimuli for apical stimulation of a selected taste bud. Stimuli were ejected for 1 s with air pressure (3.5 psi; Pressure System IIe, Toohey Company, Fairfield, NJ, USA). Different pipettes were mounted for each taste stimulus. All stimulus solutions contained 2 µM fluorescein to monitor stimulus application, duration and concentration.

CGD-loaded taste cells were viewed with a laser-scanning confocal microscope, using an argon laser (Fluoview FV-300, Olympus, Tokyo, Japan). Images were captured at 1.1 s intervals. Fluorometric signals are expressed as relative fluorescence change: Δ*F/F* = (*F–F_0_*)/*F_0_*, where *F_0_* denotes the resting fluorescence level corrected for any bleaching that occurred during the recording. Using Δ*F/F* corrects for variations in baseline fluorescence, cell thickness, total dye concentration and illumination [49]. Peak Δ*F/F* constituted the response amplitude for statistical quantification.

### Data analysis

Statistical analyses using paired Student's *t*-tests were applied to determine whether the changes in the response amplitudes (peak Δ*F/F)* to a given treatment were significant. Data presented in bar graphs show mean ± SEM.

### Reagents and solutions

γ-glutamyl-valinyl-glycine (γEVG) was synthesized by Kokusan Chemicals (Tokyo, Japan). Cinacalcet, NPS2143 and SC45647 were chemically synthesized in our facility by previously described methods [50]–[52]. All other chemicals including monopotassium L-glutamate (MPG) were purchased from Sigma Chemical (St Louis, MO, USA). All tastants were freshly dissolved in Tyrode's solution for each experiment. The standard medium consisted of Tyrode's solution, which is composed of, in mM: 135 NaCl, 5 KCl, 1.5 CaCl_2_, 1 MgCl_2_, 10 HEPES, 10 glucose, 10 sodium pyruvate and 5 NaHCO_3_, pH 7.2; 318–323 mOsm. For Ca^2+^-free Tyrode's solution, CaCl_2_ was removed, and 0.2 mM EGTA was added.

### Immunohistochemistry

Mouse circumvallate tissues were fixed in 4% paraformaldehyde and cryoprotected in 10–30% sucrose for 3.5 h at 4°C. Frozen sections (12 µm) were prepared and blocked in Protein Block (Dako, Glostrup, Denmark) with 1% Triton X-100 (Sigma) for 45 min at room temperature. Sections were then incubated for 1 h with a primary antibody [rat anti-CaSR, diluted 1∶400; rabbit anti-PLCβ2, 1∶500 (sc-206, Santa Cruz, Santa Cruz, CA, USA); rabbit anti-NCAM, 1∶400 (AB5032, Millipore, Billerica, MA, USA) and rabbit anti-T1R3, 1∶800 (kind gift from Dr. Iwatsuki, Ajinomoto Co., Inc.) [53]], followed by a secondary antibody (1∶1000): Alexa Fluor 488 labeled donkey anti-rat IgG antibody for anti-CaSR (A-21208, Invitrogen) and Alexa Fluor 568 labeled goat anti-rabbit IgG antibody for anti-PLCβ2, anti-NCAM and anti-T1R3 (A-11036, Invitrogen). CaSR was detected using a specific antibody generated in our lab (host = rat). The anti-mouse-CaSR antibody recognizes the protein sequences: KSNSEDPFPQPERQKQQ, QGPMVGDHQPEIESPDE and MRQNSLEAQKSNDTLNR, corresponding to residues 917–933, 1034–1050 and 994–1010 of the mouse CaSR, respectively. Negative controls without the primary antibody were processed in parallel in every experiment. Images were obtained with a laser-scanning confocal microscope (Olympus). We estimated a thickness of ∼3 µm for the optical section taken by the confocal microscope.

### RT-PCR analysis

RT-PCR amplification was performed using primers that amplify the mouse *CaSR*, *PLCβ2*, *Snap25* and *β-actin*. Briefly, dissected tongue containing circumvallate and foliate papillae was injected into the submucosal layer with a mixture of 1 mg/ml collagenase A (Roche Applied Science, Indianapolis, IN, USA), 2.5 mg/ml Dispase II (Roche Applied Science) and 1 mg/ml trypsin inhibitor (Sigma), and then incubated for 20 min at room temperature. The papilla-containing epithelium was peeled from the underlying connective tissue. Total RNA was isolated from the epithelial papillae and from the epithelium without taste buds (RNA micro kit, Agilent Technologies, Santa Clara, CA, USA). Purified RNA was denatured, and first-strand cDNA was synthesized using oligo(dT)_12–18_ primer and reverse transcriptase (Super Script III, Invitrogen). cDNA was used as a template in a 20 µl PCR mixture with Taq polymerase (Invitrogen). PCR conditions were as follows: 94°C for 2 min, followed by 29–35 cycles of 94°C for 30 s, 58°C for 20 s, and 72°C for 45 s. The PCR products were analyzed by gel electrophoresis with GelRed staining (Biotium, Hayward, CA, USA). The primers used were as follows: *CaSR*, 5′-tcgagaccccttacatggac-3′ (forward) and 5′-agtagttccccaccaggtca-3′ (reverse); *PLCβ2*, 5′-ctcgctttgggaagtttgc-3′ (forward) and 5′-gcattgactgtcatcgggt-3′ (reverse); *Snap25*, 5′-ggcaataatcaggatggagtag-3′ (forward) and 5′-agatttaaccacttcccagca-3′ (reverse); *β-actin*, 5′-caccctgtgctgctcacc-3′ (forward) and 5′-gcacgatttccctctcag-3′ (reverse).

## Supporting Information

Figure S1 **Human and mouse CaSR have similar properties for** ***kokumi*** **substances.** Concentration-response curves for cinacalcet (A), glutathione (GSH; B) and γ-glutamyl-valinyl-glycine (γEVG; C) in human (filled) or mouse (open) CaSR-expressing HEK cells. For the tested CaSR agonists, we observed very similar EC_50_ values in both species of CaSR. The EC_50_ values for cinacalcet, GSH and γEVG were 0.207, 0.058 and 0.033 µM for human CaSR, and 0.580, 0.058 and 0.032 µM for mouse CaSR, respectively.
(TIF)

Method S1 **Determination of CaSR activity using HEK293 cells.** Full-length human and mouse CaSR cDNA were isolated by RT-PCR and validated by sequencing. Both constructs were cloned into pcDNA3.1 (Invitrogen) for functional experiments. HEK cells were transfected in parallel using Fugene 6 (Roche, Indianapolis, IN, USA) with constructs of human- or mouse-CaSR, or with the empty expression vector, pcDNA3.1. After 24 h, cells were harvested and seeded in a 96-well plate. Cells were loaded with the calcium indicator dye, Calcium 3 (Molecular Devices, Sunnyvale, CA, USA), and responses were measured with FRIPR or FLEX Station (Molecular Devices).
(DOC)

## References

[1] Brown EM, Gamba G, Riccardi D, Lombardi M, Butters R (1993). Cloning and characterization of an extracellular Ca^2+−^sensing receptor from bovine parathyroid.. Nature.

[2] Chattopadhyay N, Vassilev PM, Brown EM (1997). Calcium-sensing receptor: roles in and beyond systemic calcium homeostasis.. Biol Chem.

[3] Brown EM, MacLeod RJ (2001). Extracellular calcium sensing and extracellular calcium signaling.. Physiol Rev.

[4] Bystrova MF, Romanov RA, Rogachevskaja OA, Churbanov GD, Kolesnikov SS (2010). Functional expression of the extracellular-Ca^2+^-sensing receptor in mouse taste cells.. J Cell Sci.

[5] San Gabriel A, Uneyama H, Maekawa T, Torii K (2009). The calcium-sensing receptor in taste tissue.. Biochem Biophys Res Commun.

[6] Ninomiya Y, Tonosaki K, Funakoshi M (1982). Gustatory neural response in the mouse.. Brain Res.

[7] McCaughey SA, Forestell CA, Tordoff MG (2005). Calcium deprivation increases the palatability of calcium solutions in rats.. Physiol Behav.

[8] Ohsu T, Amino Y, Nagasaki H, Yamanaka T, Takeshita S (2010). Involvement of the calcium-sensing receptor in human taste perception.. J Biol Chem.

[9] Dunkel A, Koster J, Hofmann T (2007). Molecular and sensory characterization of gamma-glutamyl peptides as key contributors to the kokumi taste of edible beans (Phaseolus vulgaris L.).. J Agric Food Chem.

[10] Toelstede S, Hofmann T (2009). Kokumi-active glutamyl peptides in cheeses and their biogeneration by Penicillium roquefortii.. J Agric Food Chem.

[11] Ueda T, Yonemitsu M, Tsubuku T, Sakaguchi M, Miyajima R (1997). Flavor characteristics of glutathione in raw and cooked foodstuffs.. Bioscience, Biotechnology, and Biochemistry.

[12] Ueda Y, Sakaguchi M, Hirayama K, Miyajima R, Kimizuka A (1990). Characteristic flavor constituents in water extract of garlic.. Agricultural and Biological Chemistry.

[13] Chaudhari N, Roper SD (2010). The cell biology of taste.. J Cell Biol.

[14] Murray RG (1993). Cellular relations in mouse circumvallate taste buds.. Microsc Res Tech.

[15] Yee CL, Yang R, Bottger B, Finger TE, Kinnamon JC (2001). “Type III” cells of rat taste buds: immunohistochemical and ultrastructural studies of neuron-specific enolase, protein gene product 9.5, and serotonin.. J Comp Neurol.

[16] Kinnamon JC, Taylor BJ, Delay RJ, Roper SD (1985). Ultrastructure of mouse vallate taste buds. I. Taste cells and their associated synapses.. J Comp Neurol.

[17] DeFazio RA, Dvoryanchikov G, Maruyama Y, Kim JW, Pereira E (2006). Separate populations of receptor cells and presynaptic cells in mouse taste buds.. J Neurosci.

[18] Huang AL, Chen X, Hoon MA, Chandrashekar J, Guo W (2006). The cells and logic for mammalian sour taste detection.. Nature.

[19] Huang YA, Maruyama Y, Stimac R, Roper SD (2008). Presynaptic (Type III) cells in mouse taste buds sense sour (acid) taste.. J Physiol.

[20] Medler KF, Margolskee RF, Kinnamon SC (2003). Electrophysiological characterization of voltage-gated currents in defined taste cell types of mice.. J Neurosci.

[21] Tomchik SM, Berg S, Kim JW, Chaudhari N, Roper SD (2007). Breadth of tuning and taste coding in mammalian taste buds.. J Neurosci.

[22] Dvoryanchikov G, Tomchik SM, Chaudhari N (2007). Biogenic amine synthesis and uptake in rodent taste buds.. J Comp Neurol.

[23] Caicedo A, Jafri MS, Roper SD (2000). *In situ* Ca^2+^ imaging reveals neurotransmitter receptors for glutamate in taste receptor cells.. J Neurosci.

[24] Maruyama Y, Pereira E, Margolskee RF, Chaudhari N, Roper SD (2006). Umami responses in mouse taste cells indicate more than one receptor.. J Neurosci.

[25] Gowen M, Stroup GB, Dodds RA, James IE, Votta BJ (2000). Antagonizing the parathyroid calcium receptor stimulates parathyroid hormone secretion and bone formation in osteopenic rats.. J Clin Invest.

[26] Richter TA, Dvoryanchikov GA, Chaudhari N, Roper SD (2004). Acid-sensitive two-pore domain potassium (K2P) channels in mouse taste buds.. J Neurophysiol.

[27] Huang L, Shanker YG, Dubauskaite J, Zheng JZ, Yan W (1999). Ggamma13 colocalizes with gustducin in taste receptor cells and mediates IP_3_ responses to bitter denatonium.. Nat Neurosci.

[28] Ogura T, Kinnamon SC (1999). IP(3)-Independent release of Ca^2+^ from intracellular stores: A novel mechanism for transduction of bitter stimuli.. J Neurophysiol.

[29] Rossler P, Kroner C, Freitag J, Noe J, Breer H (1998). Identification of a phospholipase C beta subtype in rat taste cells.. Eur J Cell Biol.

[30] Zhang Y, Hoon MA, Chandrashekar J, Mueller KL, Cook B (2003). Coding of sweet, bitter, and umami tastes: different receptor cells sharing similar signaling pathways.. Cell.

[31] Bleasdale JE, Thakur NR, Gremban RS, Bundy GL, Fitzpatrick FA (1990). Selective inhibition of receptor-coupled phospholipase C-dependent processes in human platelets and polymorphonuclear neutrophils.. J Pharmacol Exp Ther.

[32] Salari H, Bramley A, Langlands J, Howard S, Chan-Yeung M (1993). Effect of phospholipase C inhibitor U-73122 on antigen-induced airway smooth muscle contraction in guinea pigs.. Am J Respir Cell Mol Biol.

[33] Thompson AK, Mostafapour SP, Denlinger LC, Bleasdale JE, Fisher SK (1991). The aminosteroid U-73122 inhibits muscarinic receptor sequestration and phosphoinositide hydrolysis in SK-N-SH neuroblastoma cells. A role for Gp in receptor compartmentation.. J Biol Chem.

[34] Conigrave AD, Quinn SJ, Brown EM (2000). L-amino acid sensing by the extracellular Ca^2+^-sensing receptor.. Proc Natl Acad Sci U S A.

[35] Wang M, Yao Y, Kuang D, Hampson DR (2006). Activation of family C G-protein-coupled receptors by the tripeptide glutathione.. J Biol Chem.

[36] Li X, Staszewski L, Xu H, Durick K, Zoller M (2002). Human receptors for sweet and umami taste.. Proc Natl Acad Sci U S A.

[37] Caicedo A, Kim KN, Roper SD (2002). Individual mouse taste cells respond to multiple chemical stimuli.. J Physiol.

[38] Michlig S, Damak S, Le Coutre J (2007). Claudin-based permeability barriers in taste buds.. J Comp Neurol.

[39] Tordoff MG, Shao H, Alarcon LK, Margolskee RF, Mosinger B (2008). Involvement of T1R3 in calcium-magnesium taste.. Physiol Genomics.

[40] Montmayeur JP, Liberles SD, Matsunami H, Buck LB (2001). A candidate taste receptor gene near a sweet taste locus.. Nat Neurosci.

[41] Nelson G, Chandrashekar J, Hoon MA, Feng L, Zhao G (2002). An amino-acid taste receptor.. Nature.

[42] Nelson G, Hoon MA, Chandrashekar J, Zhang Y, Ryba NJ (2001). Mammalian sweet taste receptors.. Cell.

[43] Huang YJ, Maruyama Y, Dvoryanchikov G, Pereira E, Chaudhari N (2007). The role of pannexin 1 hemichannels in ATP release and cell-cell communication in mouse taste buds.. Proc Natl Acad Sci U S A.

[44] Huang YJ, Maruyama Y, Lu KS, Pereira E, Plonsky I (2005). Mouse taste buds use serotonin as a neurotransmitter.. J Neurosci.

[45] Finger TE, Danilova V, Barrows J, Bartel DL, Vigers AJ (2005). ATP signaling is crucial for communication from taste buds to gustatory nerves.. Science.

[46] Dando R, Roper SD (2009). Cell-to-cell communication in intact taste buds through ATP signalling from pannexin 1 gap junction hemichannels.. J Physiol.

[47] Romanov RA, Rogachevskaja OA, Bystrova MF, Jiang P, Margolskee RF (2007). Afferent neurotransmission mediated by hemichannels in mammalian taste cells.. EMBO J.

[48] Roberts CD, Dvoryanchikov G, Roper SD, Chaudhari N (2009). Interaction between the second messengers cAMP and Ca^2+^ in mouse presynaptic taste cells.. J Physiol.

[49] Helmchen G (2000). Calibration of fluorescent calcium indicators; Yuste R, Lanni F, Konnerth A, editors..

[50] Rodriguez M, Nemeth E, Martin D (2005). The calcium-sensing receptor: a key factor in the pathogenesis of secondary hyperparathyroidism.. Am J Physiol Renal Physiol.

[51] Rybczynska A, Lehmann A, Jurska-Jasko A, Boblewski K, Orlewska C (2006). Hypertensive effect of calcilytic NPS 2143 administration in rats.. J Endocrinol.

[52] Nofre G, Tinti JM, Chatzopoulos FO (1987). Sweetening agents.. US patent.

[53] Iwatsuki K, Ichikawa R, Hiasa M, Moriyama Y, Torii K (2009). Identification of the vesicular nucleotide transporter (VNUT) in taste cells.. Biochem Biophys Res Commun.

