# Supplementary figures and images for: Kokumi Substances, Enhancers of Basic Tastes, Induce Responses in Calcium-Sensing Receptor Expressing Taste Cells
Source: PLoS One. 2012 Apr 12;7(4):e34489. doi: 10.1371/journal.pone.0034489 (PMC3325276; doi:10.1371/journal.pone.0034489)

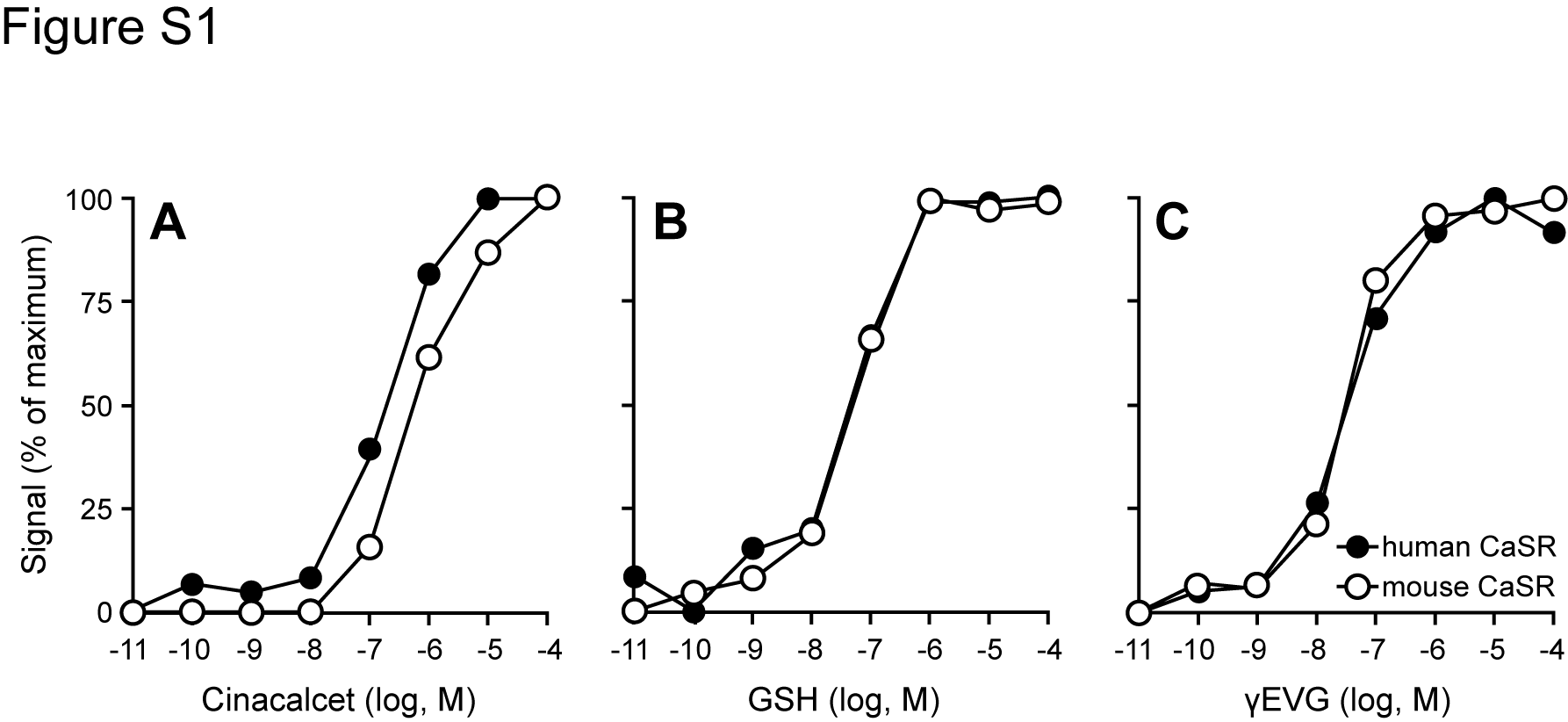

Supplement: Figure S1 — Human and mouse CaSR have similar properties for kokumi substances. Concentration-response curves for cinacalcet (A), glutathione (GSH; B) and γ-glutamyl-valinyl-glycine (γEVG; C) in human (filled) or mouse (open) CaSR-expressing HEK cells. For the tested CaSR agonists, we observed very similar EC50 values in both species of CaSR. The EC50 values for cinacalcet, GSH and γEVG were 0.207, 0.058 and 0.033 µM for human CaSR, and 0.580, 0.058 and 0.032 µM for mouse CaSR, respectively. (TIF) [file pone.0034489.s001.tif]
