# Supplementary material for: Kokumi Substances, Enhancers of Basic Tastes, Induce Responses in Calcium-Sensing Receptor Expressing Taste Cells
Source: PLoS One. 2012 Apr 12;7(4):e34489. doi: 10.1371/journal.pone.0034489 (PMC3325276; doi:10.1371/journal.pone.0034489)
Supplement: Method S1 — Determination of CaSR activity using HEK293 cells. Full-length human and mouse CaSR cDNA were isolated by RT-PCR and validated by sequencing. Both constructs were cloned into pcDNA3.1 (Invitrogen) for functional experiments. HEK cells were transfected in parallel using Fugene 6 (Roche, Indianapolis, IN, USA) with constructs of human- or mouse-CaSR, or with the empty expression vector, pcDNA3.1. After 24 h, cells were harvested and seeded in a 96-well plate. Cells were loaded with the calcium indicator dye, Calcium 3 (Molecular Devices, Sunnyvale, CA, USA), and responses were measured with FRIPR or FLEX Station (Molecular Devices). (DOC) [file pone.0034489.s002.doc]

**Supporting Information**

**Figure S1**

**Human and mouse CaSR have similar properties for *kokumi* substances.** Concentration-response curves for cinacalcet (A), glutathione (GSH; B) and γ-glutamyl-valinyl-glycine (γEVG; C) in human (filled) or mouse (open) CaSR-expressing HEK cells. For the tested CaSR agonists, we observed very similar EC_50_ values in both species of CaSR. The EC_50_ values for cinacalcet, GSH and γEVG were 0.207, 0.058 and 0.033 μM for human CaSR, and 0.580, 0.058 and 0.032 μM for mouse CaSR, respectively.

**Supplementary Methods**

*Determination of CaSR activity using HEK293 cells*

Full-length human and mouse *CaSR* cDNA were isolated by RT-PCR and validated by sequencing. Both constructs were cloned into pcDNA3.1 (Invitrogen) for functional experiments.

HEK cells were transfected in parallel using Fugene 6 (Roche, Indianapolis, IN, USA) with constructs of human- or mouse-CaSR, or with the empty expression vector, pcDNA3.1. After 24 h, cells were harvested and seeded in a 96-well plate. Cells were loaded with the calcium indicator dye, Calcium 3 (Molecular Devices, Sunnyvale, CA, USA), and responses were measured with FRIPR or FLEX Station (Molecular Devices).
